# Older people admitted to Darlinghurst Reception House in the nineteenth century

**DOI:** 10.1177/10398562261424743

**Published:** 2026-02-10

**Authors:** Brian Draper

**Affiliations:** Discipline of Psychiatry and Mental Health, School of Clinical Medicine, University of NSW, Sydney, NSW, Australia; ‘Eastern Suburbs Older Persons' Mental Health Service, Prince of Wales Hospital, Randwick, NSW, Australia

**Keywords:** old age, nineteenth century, assessment, alcohol, admissions

## Abstract

**Aims:**

To describe the clinical features and outcomes of persons aged 60 years and over admitted to Darlinghurst Reception House between 1868 and 1900, and to compare their outcomes and use of alcohol with those under the age of 60.

**Methods:**

Data were extracted from the medical case books and admission and discharge registers of Darlinghurst Reception House at the New South Wales State Archives.

**Main findings:**

There were 12,745 admissions of which 911 (7.1%) were aged 60 years and over. The majority had cognitive symptoms (54%), followed by behavioural issues (32.3%) and psychosis (19.6%). Alcohol was implicated in 129 cases (14.2%), less than in admissions aged under 60 (22.5%). Dementia was diagnosed in over 50%. Most (*n* = 721, 79.1%) were transferred to a hospital for the insane, 178 (19.5%) were discharged (38.2% alcohol-related) and 12 (1.3%) died. These outcomes were worse than for those aged under 60 in whom 28.8% were discharged (57.9% alcohol-related) and 0.7% died.

**Conclusions:**

Older people were regularly admitted to Darlinghurst Reception House for mainly cognitive disorders that usually resulted in transfer to a hospital for the insane. Alcohol was less frequently implicated than in younger people.

## Introduction

In 19th century New South Wales (NSW), all admissions to hospitals for the insane were involuntary. Under the provisions of the Dangerous Lunatics Act 1843, the legal process for admission required two medical practitioners to determine that the person was a ‘dangerous lunatic or dangerous idiot’, with the assessment being undertaken in a gaol house or public hospital.^1^

Concerns about the use of gaols for these purposes had been raised in 1854 by Dr George Walker, medical officer of Tarban Creek Asylum, when he complained to then Leader of the Opposition, Henry Parkes, about the way those pronounced insane at the Central Police Court were being treated in Darlinghurst Gaol. Apart from the delays of 3 weeks or more before transfer to Tarban Creek, Walker claimed that the patient arrived in an emaciated condition with festering sores and vermin and that while in the gaol, they were often treated violently and robbed of their rations. Walker felt that the patients were treated as felons and that the delays increased the likelihood that they would become incurably insane. A Commission of Enquiry established by the Legislative Council in 1855, while finding no evidence to support Walker’s complaints about Darlinghurst Gaol, was critical of the facilities at Tarban Creek and its location, recommending the construction of a new asylum. Implicitly the report indicated government neglect and indifference.^2,3^

The major recommendations of the Enquiry (construction of a new asylum and moving Tarban Creek Asylum to a more suitable site) were not acted upon.^3^ In 1863, the Catholic Bishop of Hobart, Robert Willson, who was knowledgeable of asylum management, visited the NSW Lunatic Asylums (Parramatta and Tarban Creek) and wrote a highly critical letter to the NSW Governor about the overcrowded, unsatisfactory accommodation and the inability to provide an environment conducive to recovery. He strongly recommended the construction of a new asylum closer to Sydney. A Select Committee of the Legislative Assembly was established in July 1863 to enquire into and report on the present state and management of lunatic asylums. In his evidence to the Select Committee, Francis Campbell, superintendent of Tarban Creek Asylum, strongly opposed the use of gaols for the assessment of sanity and recommended the establishment of a reception house for this purpose. The intent was for a short-term admission with medical review to determine the individual’s need for admission to a hospital for the insane. The Select Committee accepted this suggestion and eventually the construction of the Darlinghurst Reception House was approved in 1866.^2,3^

Darlinghurst Reception House opened in July 1868 and under provisions of the Lunacy (Amendment) Act 1867. Justices were allowed to commit persons directly to a reception house rather than to gaol, which was the usual practice of police and Justices, either to allow assessments of the sanity of individuals by medical practitioners, or as a temporary residence for persons found to be insane and awaiting transfer to a hospital for the insane.^2,4^ It was intended that admissions would only remain in the reception house for up to 14 days.

Over the first decade the reception house admissions gradually increased to over 300 patients per year.^5^ Initially, relatively few patients were discharged, averaging between 4 and 15% annually until 1881 when the Lunacy Act was amended to allow ‘magistrates to remand cases of doubtful and temporary mental aberration to the reception house instead of to gaol’.^6^ Subsequently, after 1882 there was an increase in admissions to over 500 per year, with 20–30% being discharged, many of the discharges being alcohol-related.^7,8^ The extent to which these alcohol-related discharges applied to older admissions is unknown. In 1883, Frederic Norton Manning, the Inspector General of the Insane, noted in his annual report that the reception house ‘enables scientific treatment to be applied under favourable conditions at an early stage of the malady, and so stops a number of cases from passing into a more advanced stage, and affords temporary refuge of the most fitting kind for cases which from their very nature must go on to Hospital for further and more lengthened treatment’.^5^ ^(page 11)^

He went on to say that this was ‘all but unique’, with the only similar institution that he was aware of being in Paris.^5^ The Reception House remained in operation until 1961.^2^

The use of Darlinghurst Reception House for people aged 60 years and over has not been previously examined. In the second half of the nineteenth century, there was a marked increase in the number of older people in the hospitals for the insane in NSW and Manning understood that this was in part due to higher population-based admission rates.^9^ Thus, there was every reason to hope that the reception house might avert unnecessary older admissions.

The aims of this paper are, firstly to describe the clinical features and outcomes of patients aged 60 years and over admitted to Darlinghurst Reception House between 1868 and 1900, and secondly, to compare their outcomes and the use of alcohol with patients under the age of 60.

## Methods

The medical case books (Volumes 1–12) and Registers for Admissions and Discharges for Darlinghurst Reception House were examined for the period July 1868 to December 1900 at the NSW State Archives. All admissions aged 60 years and over were identified and the following data (where available) were extracted – date of admission, age, sex, marital status, residence in rural or regional NSW, diagnosis, implication of alcohol in the presentation, clinical notes, and disposal. For admissions under the age of 60, sex, implication of alcohol in the presentation, and disposal were extracted. Between 1882 and 1898, two Registers for Admissions and Discharges were maintained. The second register was commenced in order to cover changes from the 1881 Lunacy Amendment Act and there were some inconsistencies in the ages of admissions between the registers and the case books which were resolved by accepting the age given in two of the three sources. Deaths recorded in the case books that sometimes did not get recorded in the registers were included in the study.

These data were supplemented with data from the Annual Reports of the Inspector General of the Insane (1881–1901). The role of alcohol and the disposal of admissions were compared between the two age groups.

Ethical approval was obtained from University of NSW Human Research Ethics Committee (iRECS8282).

## Results

In the study period, there were 12,745 admissions, of which 911 (7.1%) were aged 60 years and over (range 60–90, median = 65) and who were predominately male (*n* = 654, 71.8%). Marital status was recorded in 761 admissions, of which 361 were married, 224 widowed, and 176 single. There were 20 transfers from a benevolent asylum and 11 transfers from a general hospital. Living arrangements were not recorded.

Clinical features are presented in Table 1. The clinical notes were usually scanty with only a few phrases or sentences to describe the reason(s) for admission; in a few cases there were no clinical notes. Most cases had cognitive symptoms, while behavioural concerns were present in nearly a third of admissions. Prominent psychotic symptoms included grandiose delusions, religiose delusions, persecutory delusions (particularly fears of being poisoned or injured), and hypochondriacal delusions, while some had command hallucinations to kill themselves.

**Table 1.** Clinical features of admissions aged ≥60 to Darlinghurst Reception House 1868–1900.^a^

Clinical feature	*N* (%)
Cognitive issues (e.g. dementia, imbecility, disorientation, idiocy, confusion, impaired memory/intellect, and poor comprehension)^b^	492 (54.0%)
Behavioural issues^c^	294 (32.3%)
Restless/wandering	115 (12.6%)
Noisiness	103 (11.3%)
Violent or dangerous	95 (10.4%)
Suicidal behaviour	73 (8.0%)
Food refusal/poor food intake	66 (7.2%)
Psychosis (delusions and/or hallucinations)	179 (19.6%)
Feeble/weak	98 (10.8%)
Rambling, incoherent speech	83 (9.1%
Sleep disturbance	56 (6.1%)
Depressed, sad, melancholy	41 (4.5%)
Incontinence (‘dirty habits’)	40 (4.4%)
Shaky	22 (2.4%)
Unable to self-care/helpless	13 (1.4%)

^a^Data extracted from the medical case books of Darlinghurst Reception House volumes 1–12, the Registers of Admissions and Discharges, and the Registers of Admissions and Discharges under Lunacy Amendment Act.

^b^’Imbecile’ and ‘Idiot’ were terms used in the 19th Century to denote cognitive impairment.

^c^Multiple behaviours were reported in some admissions.

Many were in poor health, being weak or feeble, with some being incontinent of urine and/or faeces, and others unable to self-care. Four cases were admitted in an obviously dying state. Few cases had specific physical illnesses identified, with severe visual impairment (*n* = 10), paralysis (*n* = 7), and a history of fits (*n* = 7) being most prominent. Most of the admissions described as ‘shaky’ were in alcohol withdrawal.

Formal diagnoses were made in 767 cases (84.2%), with 11 determined to be sane (see Table 2). The types of diagnoses used varied over time, for example, monomania was used between 1868 and 1885, delusional melancholia from 1886 onwards, and delusional insanity from 1896 onwards. Putative causes of the mental disorders were provided in a minority of cases with alcohol (*n* = 129, 14.2%) the most common, though less frequent than in admissions of those under age 60 where it was implicated in 2667 cases (22.5%). Of the alcohol-related cases, only 4.6% were aged 60 years and over, being implicated in 19 cases of dementia. Other causes in those aged ≥60 included old age/senility (*n* = 46, 5.0%), sunstroke/exposure (*n* = 18, 2.0%), head injury (*n* = 17, 1.9%), business/domestic troubles (*n* = 14, 1.5%), pecuniary/property losses (*n* = 10, 1.1%), and family deaths (*n* = 5, 0.5%). While General Paralysis of the Insane (GPI) was diagnosed in three cases and tabes dorsalis in a fourth case of dementia, syphilis was not mentioned.

**Table 2.** Formal psychiatric diagnoses.^a^

Diagnosis	*N* (%)
Dementia (including acute, chronic, and senile)	462 (50.7%)
Melancholia (including acute, chronic, religious, and delusional)	103 (11.3%)
Alcohol (including delirium tremens, alcoholism, intemperance, and alcoholic insanity)	85 (9.3%)
Mania (including acute, subacute, chronic, religious, and epileptic)	64 (7.0%)
Monomania or delusional insanity	50 (5.5%)
General paralysis of the insane	3 (0.3%)
No formal diagnosis – sane	11 (1.2%)
No diagnosis	133 (14.6%)

^a^Data extracted from the medical case books of Darlinghurst Reception House volumes 1–12, the Registers of Admissions and Discharges, and the Registers of Admissions and Discharges under Lunacy Amendment Act.

Treatments offered were largely supportive. Those in alcohol withdrawal were usually provided with bed rest and sometimes brandy until they improved. The weak and feeble were usually given beef tea and wine or brandy. Restraints with muffs and seclusion were used infrequently in those who were dangerous. Chloral hydrate and bromides were used for sedation. Force feeding in food refusal was uncommon.

Outcomes of admissions are summarised in Table 3. The lower discharge rates in those aged ≥60 is largely due to the lower number of older people with alcohol issues as only 68 (38.2%) of discharges of those aged ≥60 related to alcohol withdrawal, while 1974 (57.9%) of discharges under age 60 related to alcohol withdrawal. It was notable that for many who lived in rural areas, the admission to the Reception House was not intended for formal assessment, but rather as a holding ward until a bed in a hospital for the insane was found. These cases had few clinical details.

**Table 3.** Age and outcomes of admission to Darlinghurst reception house 1868–1900.^a^

Outcome	Aged <60 (*N* = 11834)	Aged ≥60 (*N* = 911)
Transferred to hospital for the insane	8340 (70.5%)	721 (79.1%)
Discharged	3409 (28.8%)	178 (19.5%)
Died	85 (0.7%)	12 (1.3%)

^a^Data extracted from the medical case books of Darlinghurst Reception House volumes 1–12, the Registers of Admissions and Discharges, and the Registers of Admissions and Discharges under Lunacy Amendment Act.

## Discussion

This study has found that people aged 60 years and over were admitted to Darlinghurst Reception House in the nineteenth century at a rate (7.1%) similar to the proportion of older people in the general population aged 15 and over that increased from 6.8% in 1871 to 8.5% in 1901.^10^ The outcomes of older admissions, however, were worse than for those under age 60 on the parameters of discharges and deaths.

Broadly the three main overlapping reasons for admission related to cognitive impairment, behavioural disturbance, or psychosis. Despite the limited information available in the clinical notes, the severity of the mental disorders for many of the individuals was quite apparent. However, many of the transfers from rural locations had little information apart from a diagnosis of dementia and often described as ‘quiet and well-conducted’ yet were almost invariably forwarded to a hospital for the insane. Such cases from metropolitan Sydney might well have been directed by the magistrate to an asylum for the aged and destitute (benevolent asylum).

The lower number of presentations with alcohol-related syndromes, mostly types of withdrawal that resolved within a week and allowed discharge, was partly responsible for the lower discharge rates. Yet over a third of older discharges were associated with alcohol issues, and alcohol was the most frequently identified putative cause of mental disorders in the older population at a rate similar to that reported (13.7%) in admissions of patients aged 60 years and over to Callan Park and Gladesville (formerly Tarban Creek) Hospitals for the Insane between 1849 and 1905, although the rates of alcohol use disorder found in this study (9.3%) is higher than reported in the hospitals for the insane (4.4%).^11^ Unlike in younger age groups, alcohol misuse by older people in nineteenth century Australia has not had much attention and these data suggest that while the impact might be less than in younger adults, it remained a major factor in precipitating mental disorders.

While there was little information about the specific physical health issues in these admissions, the large number who were feeble, weak, or dying suggests that many had significant health issues. That the majority were either widowed or single suggests it is likely that they had limited support in the community. None were transferred to a general hospital as in this era ‘incurable’ disorders would not likely be accepted for admission and the general supportive care required could be provided in the reception house. This did not mean that such admissions to the reception house were felt to be appropriate and some who stabilized were discharged via the Police Court (the legal process most frequently used from 1882 onwards) and are likely to have been referred by the magistrate to an asylum for the destitute and aged, a process well-established for older people without family support.^12^

Those who were discharged because they were determined to be sane were generally quiet, well-behaved individuals though not necessarily free of a mental disorder, for example, one man was making rambling statements about his late wife’s infidelity and a second man believed he possessed ‘millions of money’. Much like in current use of mental health legislation, the issue was whether there were sufficient grounds for involuntary detention.

Clinical diagnoses in nineteenth century NSW have a variable correlation with current diagnostic practice. An examination of diagnostic practices in older admissions to Callan Park and Gladesville Hospitals between 1849 and 1905 found that nearly two thirds of dementia diagnoses in that era could be regarded as consistent with a 21st century diagnosis of dementia, just over half of mania diagnoses could be regarded as mania currently, while nearly 90% of melancholia/delusional melancholia diagnoses were 21st century major depression diagnoses. There was a broad range of differential diagnoses. Interestingly, the diagnoses of monomania and delusional insanity that were made in the reception house were not apparent in the hospitals for the insane where ‘delusional mania’ was used for similar cases.^11^ Overall, the majority are likely to have had a form of dementia and in this era, hospitals for the insane were the main setting for long term residential care particularly in those with any semblance of behavioural disorders. The relatively few cases of GPI is consistent with the findings in this age group in the hospitals for the insane^11^; GPI was regularly diagnosed in dementia cases under the age of 60 as noted in a study of young onset dementia in 1891.^13^

## Conclusion

Older people were admitted to Darlinghurst Reception House at rates consistent with numbers in the community though had higher mortality and lower discharge rates than younger adults, the latter largely due to lower rates of alcohol withdrawal syndromes. That nearly 20% of older admissions were discharged suggests that it achieved the aim of reducing unnecessary older admissions.

## Data Availability

Data is available from the corresponding author upon request.*
